# Modeling the Action Potential in Characeae *Nitellopsis obtusa*: Effect of Saline Stress

**DOI:** 10.3389/fpls.2019.00082

**Published:** 2019-02-18

**Authors:** Vilma Kisnieriene, Indre Lapeikaite, Vilmantas Pupkis, Mary Jane Beilby

**Affiliations:** ^1^Department of Neurobiology and Biophysics, Life Sciences Center, Institute of Biosciences, Vilnius University, Vilnius, Lithuania; ^2^School of Physics, The University of NSW, Sydney, NSW, Australia

**Keywords:** Characeae, action potential across plasma and tonoplast membranes, Thiel-Beilby model, second messengers, saline stress

## Abstract

Action potentials (AP) of characean cells were the first electrical transients identified in plants. APs provide information about plethora of environmental cues. Salinity stress is critical for plants and impacts on excitability. The AP of brackish Characeae *Nitellopsis obtusa*, obtained in artificial pond water (APW) and under osmotic stress of 90 or 180 mM sorbitol APW or saline stress of 50 or 100 mM NaCl APW, were simulated by the Thiel-Beilby model (Beilby and Al Khazaaly, 2016). The model is based on a paradigm from animal systems, featuring the second messenger inositol 1,4,5-triphosphate (IP_3_) mediating the opening of Ca^2+^ channels on internal stores. In plants the IP_3_ receptors have not been identified, so other second messengers might translate the threshold plasma membrane depolarization to Ca^2+^ release. The increased Ca^2+^ concentration in the cytoplasm activates Cl^−^ channels, which lead to the depolarizing phase of the AP. The repolarization to normal resting potential difference (PD) results from the Ca^2+^ being re-sequestered by the Ca^2+^ pumps, the closure of the Cl^−^ channels, efflux of K^+^ through the depolarization-activated outward rectifier channels and the continuing activity of the proton pump. The *Nitellopsis* AP form is longer in APW compared to that of *Chara*, with more gradual repolarization. The tonoplast component of the AP is larger than that in *Chara australis*. The plasma membrane AP is prolonged by the exposure to saline to a “rectangular” shape, similar to that in *Chara*. However, the changes are more gradual, allowing more insight into the mechanism of the process. It is possible that the cells recover the original AP form after prolonged exposure to brackish conditions. Some cells experience tonoplast APs only. As in *Chara*, the proton pump is transiently inhibited by the high cytoplasmic Ca^2+^ and gradually declines in saline media. However, if the cells are very hyperpolarized at the start of the experiment, the pump inhibition both by the AP and by the saline medium is mitigated. The model parameters and their changes with salinity are comparable to those in *Chara*.

## Introduction

*Nitellopsis obtusa* is a slightly salt tolerant Characeae that can grow at salinities of ~50 mM NaCl and survives transient exposures to 100 mM NaCl or more (Winter et al., 1999). *Nitellopsis* cells have not mastered the turgor regulation by importing (or exporting) Cl^−^ and K^+^, as seen in *Chara longifolia* (Hoffmann and Bisson, 1986) and *Lamprothamnium* (Bisson and Kirst, 1980), which enables these Characeae to survive high salinities and rapid salinity changes (Hoffmann and Bisson, 1990; Beilby and Shepherd, 1996; Al Khazaaly and Beilby, 2007). However, upon increased salinity in the medium, *Nitellopsis* elevates partial internal osmotic pressure by increasing Na^+^ and sucrose concentrations in the vacuole (Winter et al., 1999).

We became interested in the action potential (AP) form of *Nitellopsis*, as Shepherd et al. (2008) found that spontaneous APs contributed to saline pathology of *Chara australis* by depleting the cells of K^+^ and Cl^−^. In this salt sensitive Characeae, a mild salinity of 50 mM NaCl had a profound effect on the AP shape and extended the AP duration from 2 to up to 60 s. The AP form was fitted by Thiel-Beilby model (Beilby and Al Khazaaly, 2016, 2017), which suggested that the re-sequestering of Ca^2+^ into internal stores was affected by salinity, leading to longer opening of the Ca^2+^-activated Cl^−^ channels. As *Nitellopsis* survives in 20–50 mM NaCl media, our present experiments set out to discover if the AP form is more resistant to salinity stress.

The Thiel-Beilby model extends the work by the Thiel group. They performed measurements of [Ca^2+^]_cyt_ changes and the voltage clamp Cl^−^ currents at the time of excitation (Biskup et al., 1999; Wacke and Thiel, 2001; Wacke et al., 2003). Their model is based on a paradigm from animal systems, where the rise of [Ca^2+^]_cyt_ is mediated by second messenger inositol 1,4,5-triphosphate (IP_3_) (Othmer, 1997). The IP_3_ signaling in plants is controversial (Munnik and Vermeer, 2010), but there is increasing experimental evidence connecting transient concentration increases of IP_3_ and cytoplasmic Ca^2+^ in circadian rhythms and upon exposure to range of abiotic stresses in higher plants (Krinke et al., 2007; Tang et al., 2007). Further, IP_3_ and DAG (diacylglycerol, formed at the same time as IP_3_) can be phosphorylated in plants to form IP_6_ and phosphatidic acid, which may also act as second messengers (Mikami, 2014).

Some aspects of the Thiel experiments could not be replicated by Tazawa and Kikuyama (2003), but it is clear that most of the [Ca^2+^]_cyt_ increase at the time of the Characeae AP comes from internal stores (Kikuyama et al., 1993) and there must be a second messenger mediating the plasma membrane potential difference (PD) decrease to a threshold level and the opening of the stores Ca^2+^ channels. By testing the model in a range of Characeae exposed to different stresses, we aim to learn more about the signaling sequences involved, as well as strategies for salt stress survival. Both *Chara* and *Nitellopsis* already provide excellent test systems for a range of biologically active compounds (Beilby and Casanova, 2014; Kisnieriene et al., 2018).

**Figure 1:**
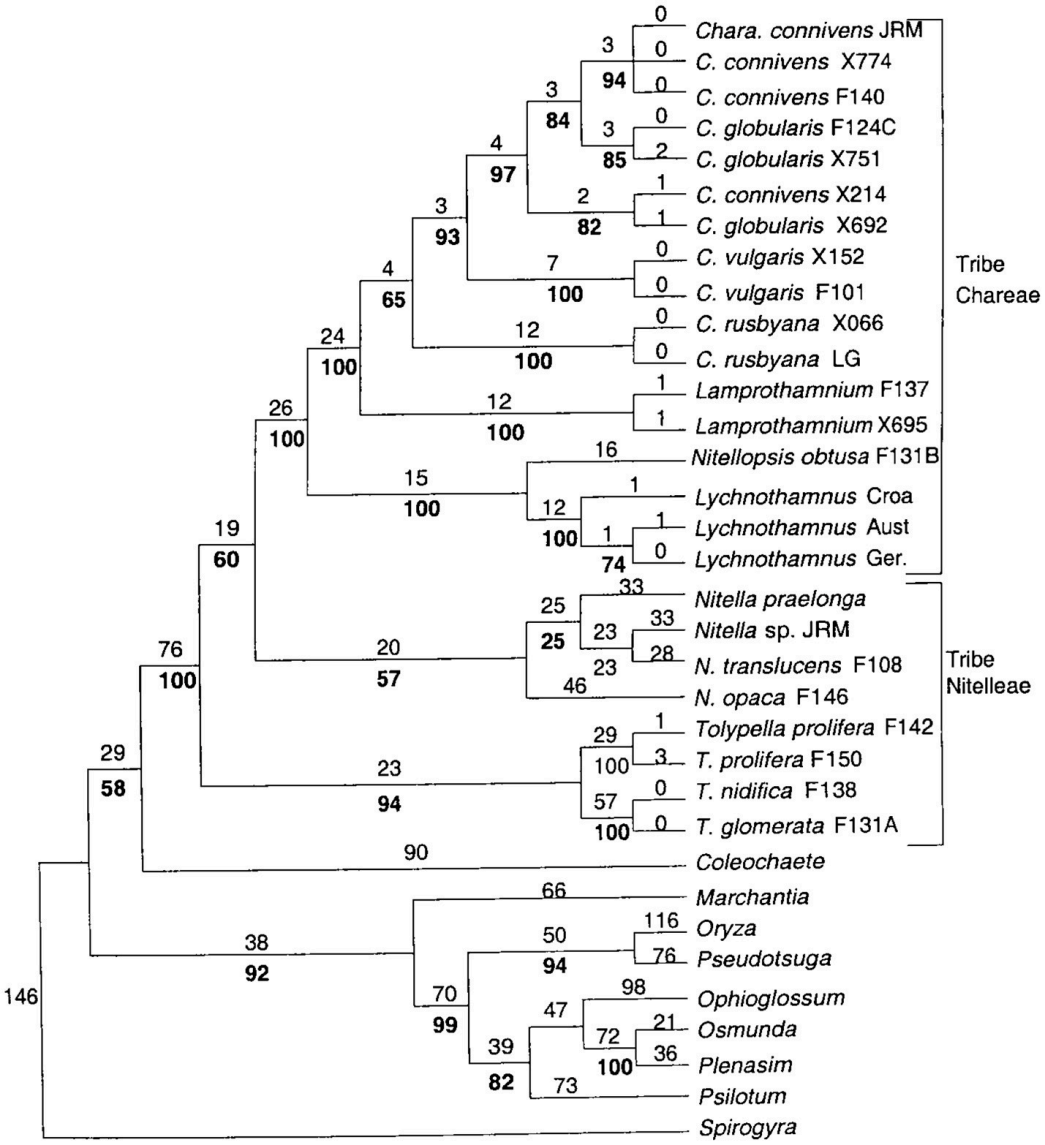
Detailed phylogenetic tree of the Characeae with branching to other charophytes and land plants. For details see Figure 1 of McCourt et al. (1999).

*Nitellopsis obtusa* constitutes a single-species genus in the tribe of *Lychnothamnus* and *Nitellopsis* (McCourt et al., 1999, see Figure 1). In this study we compare the short term (hours) salinity responses of the *Nitellopsis* AP to those of salt sensitive *Chara australis*. We found that the *Nitellopsis* AP undergoes similar changes, but these are more gradual and can be alleviated if the proton pump remains active, keeping the cell resting PD more negative. In future studies we will follow the electrophysiology of the long time survival (weeks to months) of *Nitellopsis* plants in brackish media. We also plan to compare the results to salt tolerant turgor regulator *Lamprothamnium*, which does not show spontaneous excitation upon salinity increase (Al Khazaaly and Beilby, 2007). With the publication of *Chara braunii* genome (Nishiyama et al., 2018), it will be possible to correlate the electrophysiological findings to structure and genetics of the ion transporters from the Characeae, chlorophyte algae and land plants.

## Materials and Methods

### Experimental Techniques

*Nitellopsis obtusa* (N.A. Desvaux) J. Groves algae were collected from Lithuania lakes during autumn months and maintained at the room temperature in glass aquariums under daylight conditions (9.5 ± 0.19 μmolm^−2^s^−1^) with light/dark photo regime of 12/12 h. The internodal cells (second or third below the tip), separated from neighboring cells were used in experiments. The internodes were kept at least overnight in buffered artificial pond water (APW) in standard light conditions.

Solutions are shown in Table 1. All chemicals were of analytical grade and were purchased from Sigma Aldrich (Lithuania).

**Table 1:** Conditions and media.

	**Cytoplasm**	**APW**
K^+^	80	0.1
Na^+^	10	1.0
Cl^−^	60	1.3
Ca^2+^	0.01	0.1
Buffer		5 mM TRIS/HEPES
pH	7.5	7.2

*Temperature: 21 ± 1°C*.

*Concentrations/mM*.

The cytoplasmic concentrations are based on Katsuhara and Tazawa (1986) measurements. For saline media 100 or 50 mM NaCl was added to APW, while 90 or 180 mM Sorbitol was added for the osmotic component only.

At the time of the experiment the cells were placed in borosilicate chamber consisting of three compartments filled with APW and were continuously bathed in a flowing solution of APW or test solution at a rate of ~1 ml/min using perfusion system Scientifica PPS (UK).

Intracellular and reference electrodes were placed in the central compartment of 0.5 cm length which was isolated from adjacent compartments with vaseline and constantly perfused. Calomel (Hg/HgCl) half-cells saturated with KCl were used in our experiments. Intracellular microelectrodes with 1 μm tip diameter were made from borosilicate glass capillaries (Kwik-Fill, World Precision Instruments Inc., USA), using high-quality micropipette puller SUTTER INSTRUMENTS P-97 (USA). The microelectrodes were filled with 3 M KCl. Reference electrode, immersed in the vicinity of the cell, was plugged with glass capillary filled with 3 M KCl in agar-agar. Current was injected and APs were evoked by external direct current using separate Ag/AgCl_2_ electrodes placed in each compartment. The current clamp mode was employed to record the membrane potential difference and AP. Universal computer I/O and data acquisition system: Voltage/Current Clamp amplifier TEC-10CX (USA), Digitizer Digidata 1440A, A/D board controlled by pCLAMP 10.2 software (Molecular Devices, USA). The clamped region of the cell in the central compartment was 5 mm long. Microelectrode impalement, using Micromanipulator: Eppendorf PatchMan (Germany), into the cell vacuole or cytoplasm was followed by 1.5 h resting period. The position of internal electrode was determined from the AP shape and the peak potential (see Figure 4). Only the cells with initial resting potential difference more negative than −180 mV, indicating the similar state of high H^+^- pump activity (Al Khazaaly and Beilby, 2007), were used in the experiments. APs were evoked in ramp mode by direct current increasing in 0.02 μA/s ramp rate. Once AP threshold potential, E_th_, was reached, stimulating current was ceased. APs were stimulated in 5 min intervals, but only first AP was used for analysis, as the subsequent AP exhibited longer duration than its' predecessor. In terms of the model, this finding indicated that either the cytoplasmic Ca^2+^ concentration or some channel or pump property (parameter) were not fully reset to resting state. More detailed exploration of this short time response will be used in future experiments. After 30 min of resting period different solutions were applied.

### AP Model

Wacke et al. (2003) based their calculations on Othmer (1997) model of the Ca^2+^ channels on the internal stores having four possible states: unbound (R), bound to IP_3_ (RI), bound to IP_3_ and activating Ca^2+^ molecule (RIC_+_), bound to IP_3_, and second inactivating Ca^2+^ molecule (RIC_+_C_−_). The channel conducts in RIC_+_ state and the refractory period might be caused by the long lifetime of the last state.

The rate equations for change in Ca^2+^ cytoplasmic concentration, C, and fractions of channels in each state: x_2_ (R), x_3_ (RI), x_4_ (RIC_+_), and x_5_ (RIC_+_C_−_):

(1a) $$\begin{array}{r} \text{R}+\text{I}\overset{k_{1},k_{-1}}{↔}\text{RI} \end{array}$$

(1b) $$\begin{array}{r} \text{RI}+\text{C}\overset{k_{2},k_{-2}}{↔}\text{RI}{\text{C}}_{+} \end{array}$$

(1c) $$\begin{array}{r} {\text{RIC}}_{+}+\text{C}\overset{k_{3},k_{-3}}{↔}\text{RI}{\text{C}}_{+}{\text{C}}_{-} \end{array}$$

In the Thiel-Beilby model (Beilby and Al Khazaaly, 2016, 2017) the excitation is initiated by an input of excitable amount of IP_3_, I_0_, into the cytoplasm. The IP_3_ concentration, I, then decays with time t and the time constant determined experimentally by repetitive stimulations (Wacke et al., 2003):

(2) $$\begin{array}{r} \text{I}={\text{I}}_{0}{\text{e}}^{-0.2\text{t}} \end{array}$$

Scaled Ca^2+^ concentration:

(3a) $$\begin{array}{rcl} {\text{x}}_{1} & = & \frac{\text{C}}{{\text{C}}_{0}} \end{array}$$

(3b) $$\begin{array}{rcl} {\text{C}}_{0} & = & \frac{\text{C}+\nu_{\text{r}}{\text{C}}_{\text{s}}}{1+\nu_{\text{r}}} \end{array}$$

C_0_ = average calcium concentration, C_s_ = Ca^2+^ concentration in the store, ν_r_ = ratio of endoplasmic reticulum (ER) volume to cytoplasmic volume.

(4) $$\frac{{\text{dx}}_{\text{1}}}{\text{dt}}=\lambda \left(\;\gamma_{\text{0}}+\;\gamma_{\text{1}}{\text{x}}_{\text{4}}\right)\left({\text{1-x}}_{\text{1}}\right)-\frac{{\text{p}}_{\text{1}}{\text{x}}_{\text{1}}^{\text{4}}}{{\text{p}}_{\text{2}}^{\text{4}}{\text{+x}}_{\text{1}}^{\text{4}}}$$

γ_0_ = permeability of Ca^2+^ store in absence of IP_3_, γ_1_ = density of IP_3_ activated channels, λ = 1 + ν_r_. The ratio in the Equation (4) is the Hill function, which describes the calcium conductance of the Ca^2+^ pump with scaled Hill coefficients ${\text{p}}_{1}{={\text{p}}^{\prime }}_{1}{/\text{C}}_{0}$ and ${\text{p}}_{2}{={\text{p}}^{\prime }}_{2}{/\text{C}}_{0}$ (Othmer, 1997). These coefficients can be adjusted to fit the data.

(5) $$\begin{array}{rcl} \frac{\text{d}{\text{x}}_{2}}{\text{dt}} & = & -{\text{k}}_{1}\text{I}{\text{x}}_{2}+{\text{k}}_{-1}{\text{x}}_{3} \end{array}$$

(6) $$\begin{array}{rcl} \frac{\text{d}{\text{x}}_{3}}{\text{dt}} & = & -\left({\text{k}}_{-1}+{\text{k}}_{2}{\text{x}}_{1}\right){\text{x}}_{3}+{\text{k}}_{1}\text{I}{\text{x}}_{2}+{\text{k}}_{-2}{\text{x}}_{4} \end{array}$$

(7) $$\begin{array}{rcl} \frac{\text{d}{\text{x}}_{4}}{\text{dt}} & = & {\text{k}}_{2}{\text{x}}_{1}{\text{x}}_{3}+{\text{k}}_{-3}{\text{x}}_{5}-\left({\text{k}}_{-2}+{\text{k}}_{3}{\text{x}}_{1}\right){\text{x}}_{4} \end{array}$$

(8) $$\begin{array}{rcl} \frac{\text{d}{\text{x}}_{5}}{\text{dt}} & = & {\text{k}}_{3}{\text{x}}_{1}{\text{x}}_{4}-{\text{k}}_{-3}{\text{x}}_{5} \end{array}$$

k_i_ (i = ± 1, ± 2, ± 3) are the rate constants for forward and backward transitions between the channel states. Some of these are scaled by C_0_: k_2_ = k'_2_C_0_ and k_3_ = k'_3_C_0_. With the scaling, all x_i_ range between 0 and 1. As the channels have to be in one of the four states, conservation condition applies:$\sum_{\text{k}=2}^{5}{\text{x}}_{\text{k}}=1$.

The k_i_ values are also adjusted to fit the data. The rate Equations (4–8) in x_i_ are numerically integrated to obtain the change in Ca^2+^ concentration in the cytoplasm (Wacke et al., 2003; Beilby and Al Khazaaly, 2016).

To calculate the PD transient we start from the rate of change of the membrane PD, V:

(9) $$\frac{\text{dV}}{\text{dt}}=-\frac{1}{{\text{C}}_{\text{m}}}\left[\frac{{\text{G}}_{\text{Cl,max}}\left({\text{V-E}}_{\text{Cl}}\right)}{\left(1+\frac{{\text{k}}_{\text{i}}}{{\text{k}}_{\text{a}}{\text{X}}_{1}}\right)}+{\text{I}}_{\text{p}}+{\text{I}}_{\text{orc}}+{\text{I}}_{\text{bkg}}+{\text{I}}_{\text{TRP,Ca}}\right]$$

C_m_ is the membrane capacitance (Table 2) and the terms inside the square bracket are all the currents flowing at the time of the AP. The ratio in the square brackets of Equation (9) is the Ca^2+^-activated chloride current, I_Cl_. Biskup et al. (1999) determined the rate constants k_a_ and k_i_ for the activation and inactivation of I_Cl_ by increased Ca^2+^ concentration in the cytoplasm (Table 2). G_Cl, max_ was set in the range suggested by previous modeling (Beilby and Coster, 1979).

**Table 2:** Model parameters.

**A**. ***Nitellopsis*** **fit parameters in APW, 90 mM Sorbitol APW and 50 mM NaCl APW for Cells 1–3, compared to standard** ***Chara australis*** **AP. The blank compartments indicate that the parameter values were unchanged**.									
**Parameter**	**AP**_**av**_, ***Chara*** **APW**	**AP**_**av**_ ***Nitellopsis*** **APW**	**Cell 1 APW**	**Sorbitol 90 mM 15 min**	**50 mM NaCl just on**	**50 mM NaCl 30 min**	**50 mM NaCl 60 min**	**Cell 2 50 mM NaCl overnight**	**Cell 3 50 mM NaCl overnight**
ν_r_	0.185								
γ_0_	0.1 s^−1^								
γ_1_	20.5 s^−1^								
p${}_{1^{\prime }}$	8.5 μM.s ^−1^	9.74	9.48	9.45	6.69	6.7	4.74	5.75	9.0
p${}_{2^{\prime }}$	0.035 μM	0.0197	0.014	0.0171	0.0085	0.009	0.004	0.013	0.33
C_0_	1.56 μM								
k_1_	12.0 (μM.s)^−1^								
k_−1_	8.0 s^−1^		8.0	8.0	8.0	8.0	7.8	7.8	7.8
k${}_{2}^{\prime }$	15.0 (μM.s)^−1^	14.2	14.2	14.25	14.25	14.27	14.25	14.25	14.25
k_−2_	1.65 s^−1^	1.595	1.595	1.6	1.604	1.6025	1.4	1.6	1.6
k${}_{3}^{\prime }$	1.8 (μM.s)^−1^	1.51	1.56	1.51	1.52	1.52	1.51	1.51	1.51
k_−3_	0.04 s^−1^	0.312	0.31	0.318	0.1955	0.1956	0.25	0.28	0.25
IP_3_	2.5 μM	2.1	2.1	2.0	2.0	2.0	0.9	1.8	0.15
k_a_	2 s^−1^	4.6	4.3	4.6	4.6	4.6	4.3	4.3	4.6
k_i_	2 s^−1^								
ΔCa^2+^ (period of application)	0.0355 (0.04–0.16 s)	0.009 (0.07–0.16)	0.011 (0.09–0.18)	0.008 (0.15–0.2)	0.02 (0.14–0.17)	0.009 (0.1–0.17)	0.009 (0.16–0.19)	0.008 (0.09–0.18)	0.004 (0.75–0.765)
**B. Parameters for the membrane transporters**									
Resting PD before after in mV	Not considered	−234	−232	−248	−240	−228	−190	−121	−68
		−239	−210	−218	−202	−192	−133	−121	−69
κ_oi_	140 s^−1^	80	80	140	400	350	70	13	No pump
${\text{k}}_{\text{io}}^{0}$	7,000 s^−1^	6,500	6,500	6,500	7,500	7,500	6,000	6,000	No pump
${\text{k}}_{\text{oi}}^{0}$	0.1 s ^−1^								
κ_io_	0.1 s^−1^								
G_bkg_	0.5 S.m^−2^		0.5	0.5	1.0	1.0	1.0	1.0	1.0
V_50+_	100 mV								
z_g_	1.0								
N_K_P_K_	6.5 × 10^−7^ m^3^.s^−1^								
[K^+^]_cyt_	100 mM	80	80	80	75	60	55	50	50
[K^+^]_o_	0.1 mM								
[Cl^−^]_cyt_	10 mM	60	60	60	60	60	60	60	60
[Cl^−^]_o_	1.3 mM		1.3	1.3	50	50	50	50	50
[Ca^2+^]_cyt_	0.02 μM								
[Ca^2+^]_o_	0.1 mM								
G_Cl, max_	10 S.m^−2^	4.0	5.0	7.2	80	84	20–4.1	5.0	20

To get better fit for Nitellopsis the Hill coefficient was reduced to n = 1:

$\frac{p1^{\prime }C}{p2^{\prime }+C}$ *from n = 2 in Chara model:* $\frac{p1^{\prime }C^{2}}{p{2^{\prime }}^{2}+C^{2}}$.

The proton pump current, I_p_, the background current, I_bkg_, and the outward rectifier current, I_orc_, are part of the electrophysiological makeup of the *Chara* plasma membrane (Beilby and Al Khazaaly, 2016; see also Chapter 2 of Beilby and Casanova, 2014).

The dependence of the pump current, I_p_, on membrane PD, V, is given by:

(10a) $$\begin{array}{rcl} {\text{I}}_{\text{p}} & = & {\text{z}}_{\text{p}}\text{FN}\frac{{\text{k}}_{\text{io}}\kappa_{\text{oi}}-{\text{k}}_{\text{oi}}\kappa_{\text{io}}}{{\text{k}}_{\text{io}}+{\text{k}}_{\text{oi}}+\kappa_{\text{io}}+\kappa_{\text{oi}}} \end{array}$$

(10b) $${\text{k}}_{\text{io}}{\text{= k}}_{\text{io}}^{0}{\text{e}}^{\frac{{\text{z}}_{\text{p}}\text{FV}}{2\text{RT}}}$$

(10c) $${\text{k}}_{\text{io}}{\text{= k}}_{\text{oi}}^{0}{\text{e}}^{-\;\frac{{\text{z}}_{\text{p}}\text{FV}}{2\text{RT}}}$$

where the charge transit through the pump across the membrane is characterized by the rate constants k_io_ and k_oi_, across a symmetrical Eyring barrier, F, R, T symbols have their usual meaning, z_p_ is the pump stoichiometry of 1, N is a scaling factor set to 2 × 10^−8^ and ${\text{k}}_{\text{io}}^{0}$ and ${\text{k}}_{\text{oi}}^{0}$ are defined at 0 PD (Hansen et al., 1981; Beilby and Al Khazaaly, 2016). The parameter values were adjusted to simulate the resting PD of each cell.

The background current, I_bkg_, is modeled empirically:

(11) $$\begin{array}{r} {\text{I}}_{\text{bkg}}={\text{G}}_{\text{bkg}}\left({\text{V}-\text{E}}_{\text{bkg}}\right) \end{array}$$

with the background conductance G_bkg_ independent of membrane PD. The reversal PD, E_bkg_, is taken as −100 mV (Shepherd et al., 2008). For parameter values see Table 2B.

The outward rectifier current, I_orc_, mainly due to K^+^, was simulated by the Goldmann-Hodgkin-Katz (GHK) equation, multiplied by the Boltzmann distribution of open probabilities, P_o−_ and P_o+_, to make the PD-dependence stronger (Beilby and Walker, 1996; Amtmann and Sanders, 1999):

(12) $${\text{I}}_{\text{orc}}=\frac{{\text{P}}_{\text{o+}}{\text{P}}_{\text{o-}}{\text{N}}_{\text{K}}{\text{P}}_{\text{K}}{\left(\text{zF}\right)}^{\text{2}}\text{V}({\left[\text{K}\right]}_{\text{i}}\text{-}{\left[\text{K}\right]}_{\text{o}}{\text{e}}^{\text{-}\frac{\text{zFV}}{\text{RT}}})}{\text{RT}({\text{1-e}}^{\text{-}\frac{\text{zFV}}{\text{RT}}})}$$

(13a) $${\text{P}}_{\text{o+}}=\frac{\text{1}}{{\text{1+e}}^{\text{-}\frac{{\text{z}}_{\text{g}}\text{F}({\text{V-V}}_{\text{50+}})}{\text{RT}}}}$$

(13b) $${\text{P}}_{\text{o-}}=\text{1}-\frac{\text{1}}{{\text{1+e}}^{\text{-}\frac{{\text{z}}_{\text{g}}\text{F}({\text{V-V}}_{\text{50-}})}{\text{RT}}}}$$

with z = valence of the transported ion, [K]_o_ and [K]_i_ are the K^+^ concentrations in the medium and cytoplasm, N_K_P_K_ = number of K^+^ channels and their permeability as a single parameter; z_g_ = number of gating charges, V_50−_ and V_50+_ = the half activation potentials, V_50_, at the negative and positive PDs of channel closure. The parameter values can be found in Table 2B. The total current, I_tot_, was fitted to the I/V characteristics of many characean cells under range of conditions (Beilby and Casanova, 2014).

The currents (Equations 10–13) were adjusted to fit the initial resting PD of the data and, in *Chara*, were assumed not to be changed by the excitation event. However, the present data suggest that the *Nitellopsis* proton pump responds promptly to the increase of cytoplasmic Ca^2+^ concentration. To follow the change in Resting PD before and after the AP (see Tables 2B, 3B, 4B and Figures 5, 8, 9), we manipulated the rate constant κ_oi_, which subsumes some of the voltage-independent steps of the pump cycle, such as ATP-, ADP-, inorganic phosphate- and H^+^ binding and de-binding and carrier recycling.

**Table 3:** Model parameters.

**A**. ***Nitellopsis*** **fit parameters in APW, 180 mM Sorbitol APW, and 100 mM NaCl APW for Cell 5, compared to standard** ***Chara australis*** **AP. The blank compartments indicate that the parameter values were unchanged**.							
**Parameter**	**AP**_**av**_, ***Chara*** **APW**	**AP**_**av**_ ***Nitellopsis*** **APW**	**Cell 5 APW**	**Sorbitol 180 mM 15 min**	**100 mM NaCl 15 min**	**100 mM NaCl 30 min**	**100 mM NaCl 60 min**
ν_r_	0.185						
γ_0_	0.1 s^−1^						
γ_1_	20.5 s^−1^						
p${}_{1^{\prime }}$	8.5 μM.s ^−1^	9.74	9.1	10.8	9.05	7.7	8.6
p${}_{2^{\prime }}$	0.035 μM	0.0197	0.016	0.045	0.012	0.011	0.053
C_0_	1.56 μM						
k_1_	12.0 (μM.s)^−1^						
k_−1_	8.0 s^−1^		8.1	8.1	8.1	8.1	8.1
k${}_{2}^{\prime }$	15.0 (μM.s)^−1^	14.2	14.0	14.0	14.0	14.0	14.0
k_−2_	1.65 s^−1^	1.595	1.59	1.64	1.64	3.04	3.04
k${}_{3}^{\prime }$	1.8 (μM.s)^−1^	1.51	1.51	1.2	1.2	1.2	1.2
k_−3_	0.04 s^−1^	0.312	0.374	0.36	0.36	0.62	0.62
IP_3_	2.5 μM	2.1	1.1	1.1	1.1	0.9	0.9
k_a_	2 s^−1^	4.6	5.5	5.5	5.5	5.5	5.5
k_i_	2 s^−1^						
ΔCa^2+^ (period of application)	0.0355 (0.04–0.16 s)	0.009 (0.07–0.16)	0.007 (0.01–0.14)	0.008 (0.01–0.14)	0.0089 (0.01–0.095)	0.009 (0.02–0.096)	0.01 (0.1– 0.18)
**B. Parameters for the membrane transporters**.							
Resting PD before after in mV	Not considered	−234	−253	−231	−167	−241	−203
		−239	−244	−208	−141	−235	−188
κ_oi_	140 s^−1^	80	175	85	40	950	100
${\text{k}}_{\text{io}}^{0}$	7,000 s^−1^	6,500	7,500	6,500	6,500	9,500	6,500
${\text{k}}_{\text{oi}}^{0}$	0.1 s ^−1^						
κ_io_	0.1 s^−1^						
G_bkg_	0.5 S.m^−2^		0.5	0.5	1.0	1.0	1.0
V_50+_	100 mV						
z_g_	1.0						
N_K_P_K_	6.5 × 10^−7^ m^3^.s^−1^						
[K^+^]_cyt_	100 mM	80	80	80	75	70	60
[K^+^]_o_	0.1 mM						
[Cl^−^]_cyt_	10 mM	60	60	60	60	60	60
[Cl^−^]_o_	1.3 mM		1.3	1.3	100	100	100
[Ca^2+^]_cyt_	0.02 μM						
[Ca^2+^]_o_	0.1 mM						
G_Cl, max_	10 S.m^−2^	4.0	6.3	3.4	13.5	80.0	15.0

**Table 4:** *Nitellopsis* fit parameters in APW and 100 mM NaCl APW for Cell 6, compared to standard *Chara australis* AP.

**A. The blank compartments indicate that the parameter values were unchanged**.								
**Parameter**	**AP_av_**, ***Chara*** **APW**	**AP**_**av**_ ***Nitellopsis*** **APW**	**Cell 6 APW**	**100 mM NaCl just on**	**100 mM NaCl 15 min**	**100 mM NaCl 30 min**	**100 mM NaCl 60 min**	**100 mM NaCl 90 min**
ν_r_	0.185							
γ_0_	0.1 s^−1^							
γ_1_	20.5 s^−1^							
p${}_{1^{\prime }}$	8.5 μM.s ^−1^	9.74	11.5	14.8	7.8	7.75	1.936	0.78
p${}_{2^{\prime }}$	0.035 μM	0.0197	0.0275	0.014	0.029	0.032	0.009	0.009
C_0_	1.56 μM							
k_1_	12.0 (μM.s)^−1^							
k_−1_	8.0 s^−1^		8.1	8.1	8.1	8.1	8.1	8.1
k${}_{2}^{\prime }$	15.0 (μM.s)^−1^	14.2	14.0	14.0	14.0	14.0	14.0	14.0
k_−2_	1.65 s^−1^	1.595	1.59	1.64	3.04	3.04	3.04	3.04
k${}_{3}^{\prime }$	1.8 (μM.s)^−1^	1.51	1.51	1.5	1.2	1.2	1.2	1.2
k_−3_	0.04 s^−1^	0.312	0.15	0.8	0.62	0.62	0.065	0.03
IP_3_	2.5 μM	2.1	2.1	2.1	0.7	0.81	0.86	0.86
k_a_	2 s^−1^	4.6	5.5	5.5	5.5	5.5	5.5	5.5
k_i_	2 s^−1^							
ΔCa^2+^ (period of application)	0.0355 (0.04–0.16 s)	0.009 (0.07–0.16)	0.016 (0.04–0.15)	0.03 (0.15–0.21)	0.0086 (0.02–0.0895)	0.0047 (0.02–0.15)	0.006 (0.1–0.14)	0.009 (0.15–0.25)
**B. Parameters for the Membrane Transporters**.								
Resting PD before after in mV	Not considered							
		−234	−120	−120	−89	−84	−103	−105
		−239	−115	−89	−84	−84	−105	−101
κ_oi_	140 s^−1^	80	5	12	No pump	No pump	No pump	No pump
k${}_{\text{io}}^{0}$	7,000 s^−1^	6,500	6,000	6,500	No pump	No pump	No pump	No pump
k${}_{\text{oi}}^{0}$	0.1 s ^−1^							
κ_io_	0.1 s^−1^							
G_bkg_	0.5 S.m^−2^		0.5	1.0	1.0	1.0	1.0	1.0
V_50+_	100 mV							
z_g_	1.0							
N_K_P_K_	6.5 × 10^−7^ m^3^.s^−1^							
[K^+^]_cyt_	100 mM	80	80	80	50	40	35	35
[K^+^]_o_	0.1 mM							
[Cl^−^]_cyt_	10 mM	60	60	60	60	60	60	60
[Cl^−^]_o_	1.3 mM		1.3	100	100	100	100	100
[Ca^2+^]_cyt_	0.02 μM							
[Ca^2+^]_o_	0.1 mM							
G_Cl, max_	10 S.m^−2^	4.0	1.2	30	4.0	4.0	5.0	9.0

Taking the initial κ_oi_, which was set to simulate the cell pre-excitation Resting PD, the best results were obtained by linear decrease (and sometimes subsequent increase) of the parameter producing small changes in the membrane PD. At the peak of the AP the chloride current is much larger than the pump current, so the shape of the AP was only affected toward the end of excitation. The example of such manipulation can be seen in Figure 3B, which describes modeling of the sequence of APs after the Cell 1 was exposed to 50 mM NaCl APW. In this simulation the initial concentration of the IP_3_ was held at 0, so that the model excitation did not occur, showing how these small changes in κ_oi_, propagate through the integration process of the AP. Unfortunately, there is no such technique in the experiments, to reveal the true time-course of the pump contribution to membrane PD. More information can be gathered in future by following the Resting PD and plasma membrane conductance for some minutes after the AP.

I_TRP, Ca_ approximates an initial fast transient inflow of Ca^2+^ across plasma membrane due to activation of transient receptor potential (TRP)—like channels, possibly activated by another second messenger diacylglycerol (DAG) formed at the same time as IP_3._ The I_TRP, Ca_ was simulated by I_Ca_ = G_Ca_(V–E_Ca_), turned on as a square pulse of Ca^2+^, with the timing adjusted to fit the data (Tables 2–4 and Figure 3B, that shows the corresponding I_Ca_ values in the model for the Cell 1 50 mM NaCl exposure AP sequence). As it is not known how much of this Ca^2+^ inflow reaches the store channels, a small fraction is added to x_1_ in the same time interval to increase the rate of depolarization to match the data (Beilby and Al Khazaaly, 2016, 2017).

To sum up the model: depolarization to threshold membrane PD produces two second messengers (IP_3_ and DAG in animal systems, yet to be definitely identified in plants). One of these transiently activates a channel in the plasma membrane that allows a brief inflow of Ca^2+^, while the other opens Ca^2+^ channels on internal stores. The increase of cytoplasmic Ca^2+^ activates plasma membrane Cl^−^ channels and Cl^−^ outflow depolarizes membrane PD. A further increase of cytoplasmic Ca^2+^ concentration closes Ca^2+^ channels on the stores, while Ca^2+^ pumps are restoring low cytoplasmic Ca^2+^, and membrane PD is repolarized by depolarization-activated outward rectifier channels, by diminishing Cl^−^ conductance and by the action of the proton pump, which is only slightly diminished by the transient high cytoplasmic Ca^2+^.

### Modeling Strategy

**Figure 2:**
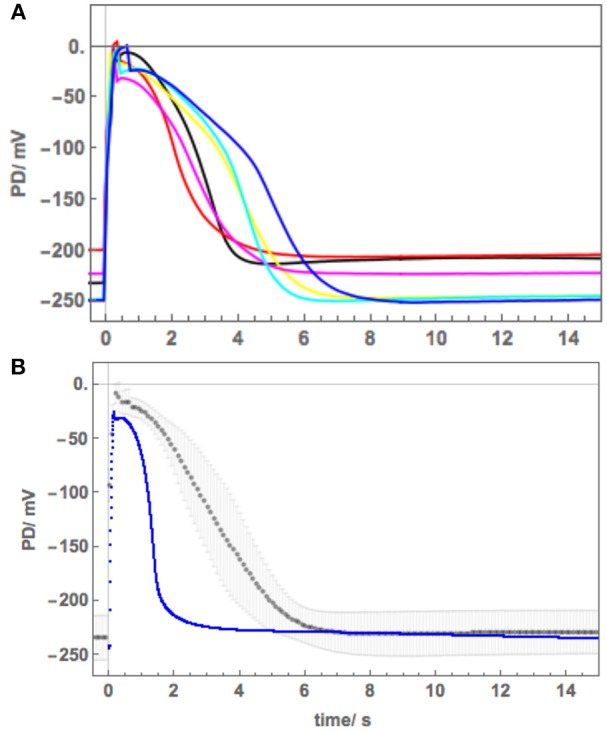
*Nitellopsis* average AP across plasma membrane **(A)** Six *Nitellopsis* APs measured across the plasma membrane from six cells in artificial pond water (APW) and at room temperature. **(B)** Computed average AP (black points) with the shaded area delimiting the standard deviations at each point. The average AP for *Chara* (blue points) under similar conditions was adapted from Beilby and Al Khazaaly (2016).

**Figure 3:**
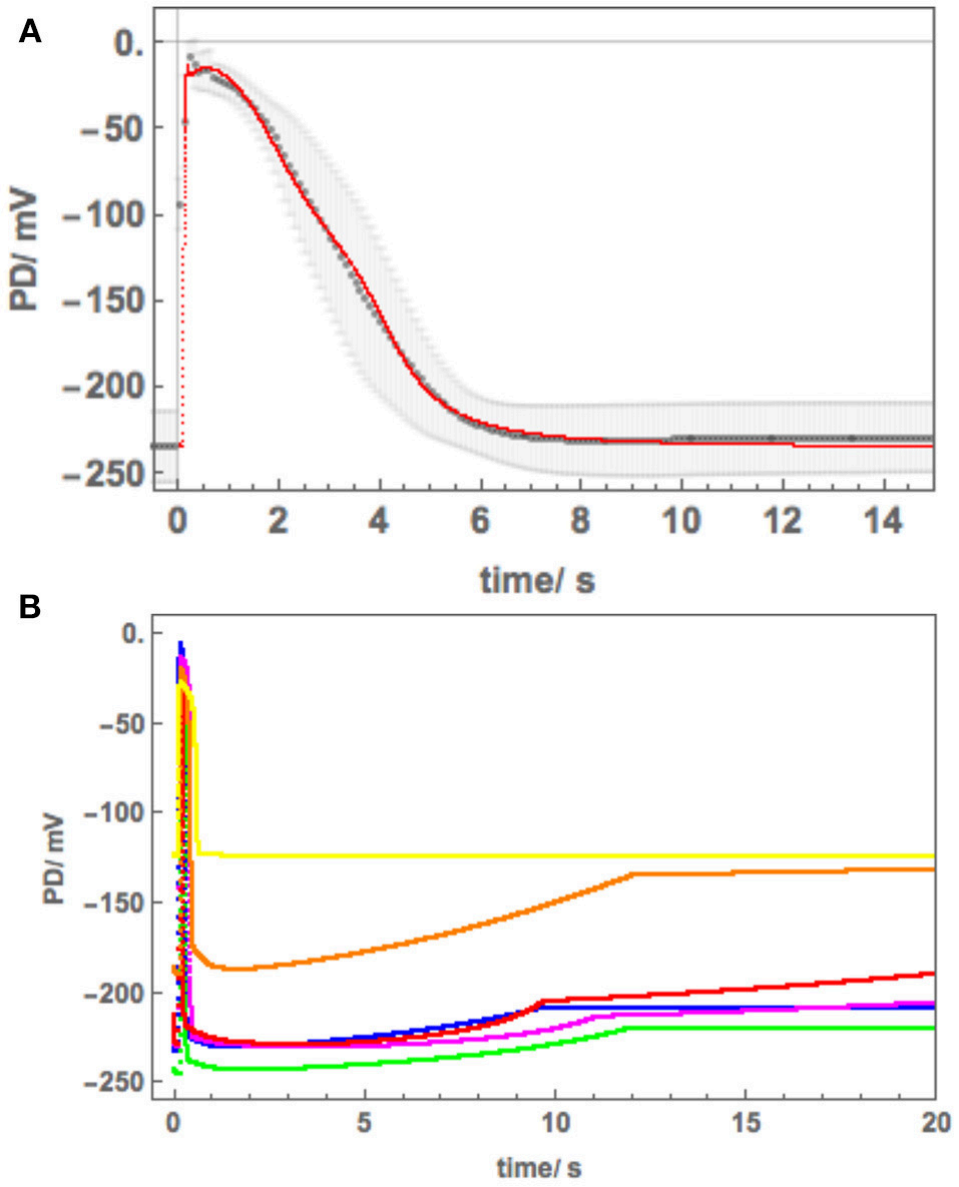
**(A)** The average AP_plasma membrane_ (black points) fitted with Thiel-Beilby model (red line). The model parameters can be seen in Table 2 and compared to those used to fit the *Chara* AP (blue line in Figure 2, Beilby and Al Khazaaly, 2016). **(B)** Modeling the proton pump inhibition by the Ca^2+^ rise at the time of the AP and saline stress in Cell 1 and 2: blue–APW, green–Sorbitol, magenta−50 mM NaCl for few min, red−50 mM NaCl for 30 min, orange−50 mM NaCl for 60 min, yellow−50 mM NaCl overnight. The pump current is diminished by the manipulation of the κ_oi_ parameter (Hansen et al., 1981).

**Figure 4:**
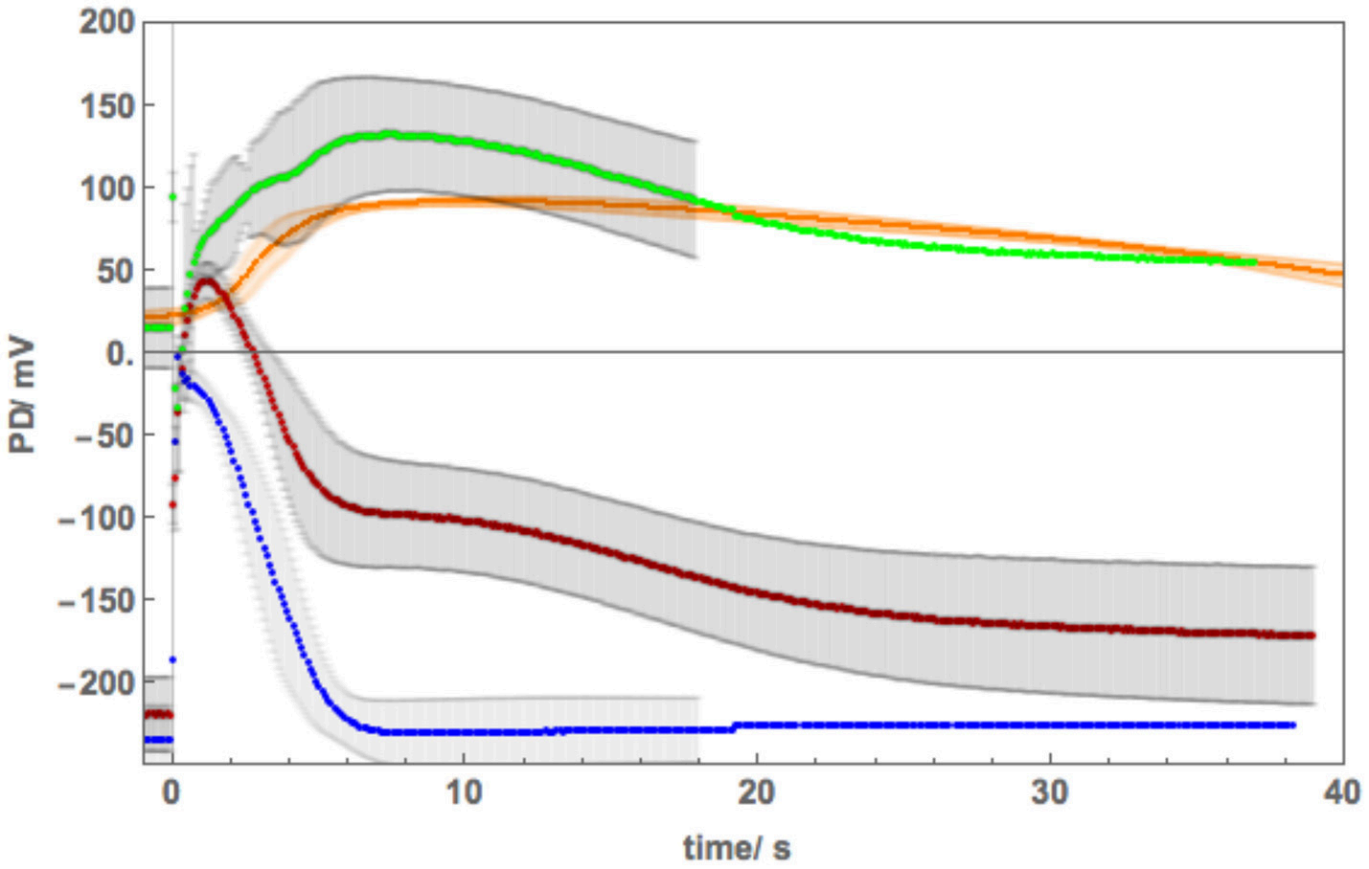
The average tonoplast AP in APW (obtained by subtraction) compared to average tonoplast AP in 100 mM NaCl. The average AP across plasma membrane (blue points) was subtracted from the average AP across both membranes calculated from 7 APs (red points). The data for the plasma membrane finished at 20 s and the steady resting PD was artificially extended, thus no standard deviation is shown after that time. The resultant tonoplast AP is shown by green points. The average saline tonoplast AP with standard deviation are depicted in orange for comparison. The data were measured from Cell 5 (see Figure 7), with the tonoplast PD estimated by adding 55 mV (approximated PD across the plasma membrane at the time of saline stress of 100 mM NaCl).

We calculated the average *Nitellopsis* AP to estimate the form variability in freshwater and to compare with the average *Chara* AP visually (Figure 2B) and in terms of the model parameters (Figure 3A, Table 2A). Initially we planned to monitor the *Nitellopsis* AP across both membranes under saline stress, as the internal PD-measuring electrode is less likely to block in long experiments. We estimated the tonoplast component of the AP by subtraction of the averaged plasma membrane component from the AP across both membranes (Figure 4). The tonoplast component amplitude and duration would have made it too difficult to distinguish the detailed changes in the plasma membrane component, especially as it is not known if the tonoplast AP also responds to saline stress. Consequently, we isolated the plasma membrane AP component by placing the PD-measuring electrode in the cytoplasm. The saline exposure in *Chara* resulted in highly variable time course of the AP changes and general membrane electrophysiology measured as current-voltage characteristics, despite selection of cells of similar size, and age from the same culture. Statistical treatment of such data had to be applied carefully, as not to obscure important features of the saline response. We were interested in the individual AP form variations: modeling these allowed us to estimate the effect of salinity on the ion transporters (Beilby and Al Khazaaly, 2009, 2016, 2017). We adopted similar approach in this study, presenting saline response time-courses of individual cells, which allowed us to model the inhibition of the proton pump by the rise in cytoplasmic Ca^2+^, the effect of the pre-excitation Resting PD dominated by different transporters, or unusual APs in Cells 3 and 4. Data from more than 20 cells with multiple APs were processed to document salinity-induced changes in AP form. Results from Cells 1–6 were chosen to illustrate (a) similarity to the response of salt-sensitive *Chara* (b) small differences that might contribute to *Nitellopsis* survival in brackish media.

## Results

### AP Form Across Plasma Membrane in Freshwater Medium

APs were excited in artificial pond water (APW, see Table 1). By placing the potential difference (PD) measuring electrode in the cytoplasm (see section Materials and Methods), the plasma membrane component of the AP was recorded. Figure 2A shows 6 plasma membrane APs from 6 cells. Note the variation in peak potential level (−29 to −4 mV), duration (4 to 7 s) and shape. In Figure 2B the averaged AP with standard deviation, shown by the shaded area, is compared to average AP at room temperature from *Chara australis* (Beilby and Al Khazaaly, 2016).

The average AP was fitted with the Thiel-Beilby model (Beilby and Al Khazaaly, 2017). The modeling started from the *Chara* parameters (Table 2A, *Chara* column). To achieve the wider *Nitellopsis* AP form, several parameters were changed (Table 2A, *Nitellopsis* column). Most parameter values were comparable to those of *Chara* model, but k_−3_, that controls the return of the Ca^2+^ channel from inactivated state (see section Materials and Methods), was an order of magnitude larger. However, good fit was only obtained when Hill coefficient n was reduced from 2 to 1 (see section Discussion). Figure 3A shows the model as a red line superimposed on the data.

### AP Components Across Plasma and Tonoplast Membranes

With the PD measuring electrode placed in the vacuole, the AP across both membranes was recorded. This impalement is much easier, as the vacuole is large and the electrode is less likely to block. Figure 4 shows average AP (computed from 7 APs) across both membranes (red points). By subtracting the average AP across plasma membrane (blue points) the average tonoplast AP was obtained (green points). The standard deviation was calculated as

$$\begin{array}{c} {\text{SD}}_{\text{tonoplast}}=\surd \left(\text{S}{\text{D}}_{\text{both membranes}}^{2}+\text{S}{\text{D}}_{\text{plasma membrane}}^{2}\right). \end{array}$$

The data for the plasma membrane AP finished at 20 s and the steady Resting PD was artificially extended, thus no standard deviations are shown after that time.

### The Effect of 50 mM NaCl on AP Across Plasma Membrane

**Figure 5:**
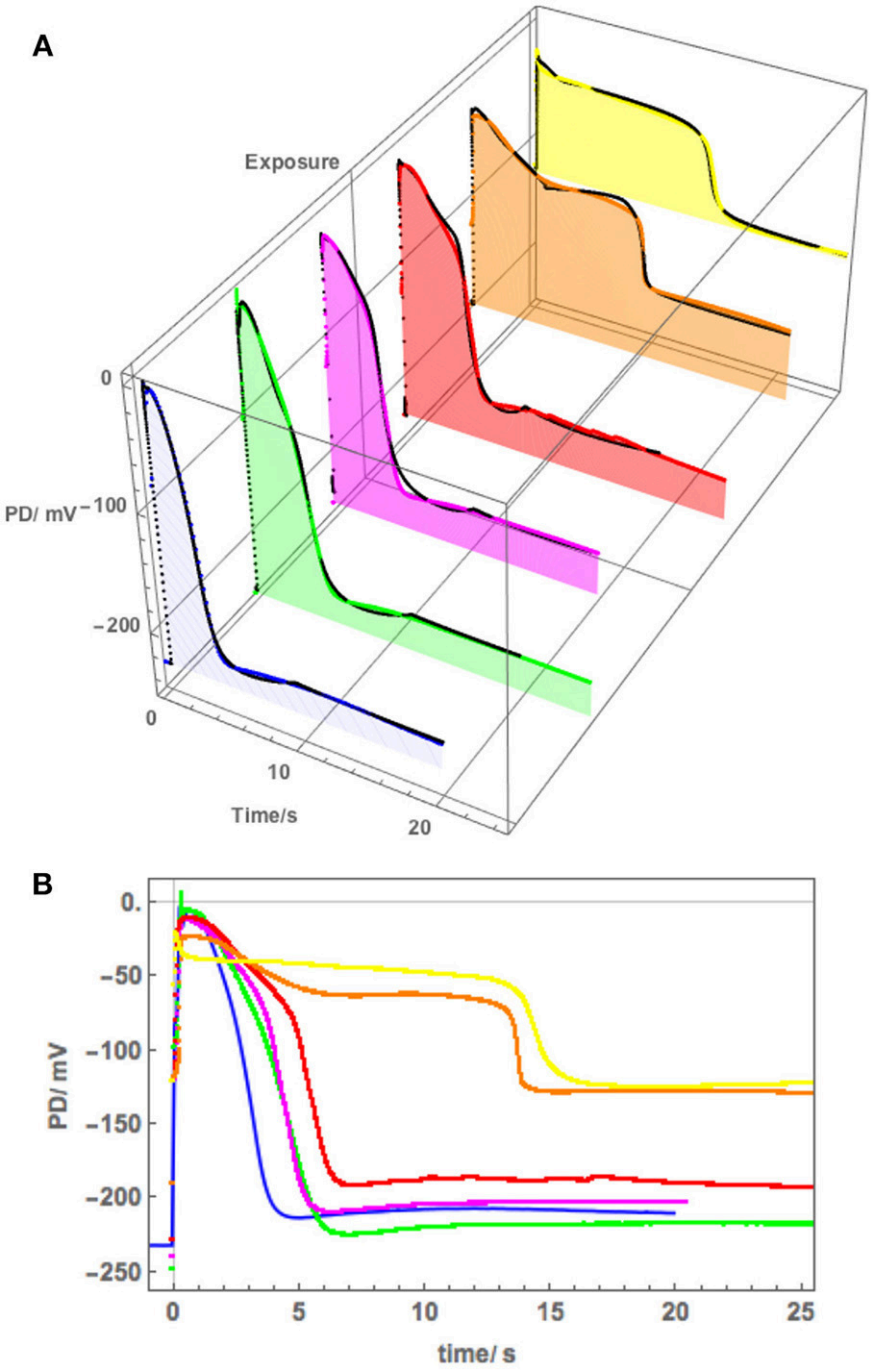
**(A)** The effect of 90 mM Sorbitol and 50 mM NaCl on the AP across the plasma membrane (Cell 1): blue–APW, green–Sorbitol APW, magenta−50 mM NaCl for few min, red−50 mM NaCl APW for 30 min, orange−50 mM NaCl APW for 60 min, yellow−50 mM NaCl APW overnight (Cell 2). The shading of each profile starts from the resting PD before excitation. Note the lower resting level after each AP, except the overnight data (yellow). The black lines show the model fit. The parameters can be found in Table 2. **(B)** Same AP sequence superimposed.

Figure 5 shows a sequence of APs, when Cell 1 was exposed to 90 mM Sorbitol APW to observe the osmotic component of the saline stress separately, followed by 50 mM NaCl APW. The 15 min exposure to Sorbitol APW slightly increased the AP duration, without greatly affecting the AP shape or the pre-excitation resting PD (compare Blue and Green curves in Figure 5B). Immediate exposure to 50 mM NaCl APW also had a slight effect (Magenta curve). After 30 min saline exposure, the AP became wider and pre-excitation resting PD depolarized slightly (Red curve). After 60 min the AP shape changed drastically (Orange curve). This AP seems to show a transition between the shape in APW to that with a prolonged flat phase from 6 to 14 s. Cell 2, which was exposed to 50 mM NaCl APW overnight, exhibited similar flat AP with a rectangular shape and low pre-excitation Resting PD near −100 mV, which was not affected by the excitation. The time course of Sorbitol/50 NaCl exposure in Cells 1 and 2 shows that *Nitellopsis* AP form is affected in similar way to *Chara*, but the process is more gradual, with the 60 min exposure (Orange curve) showing a transition from slightly extended “normal” AP to a typical rectangular saline AP.

**Figure 6:**
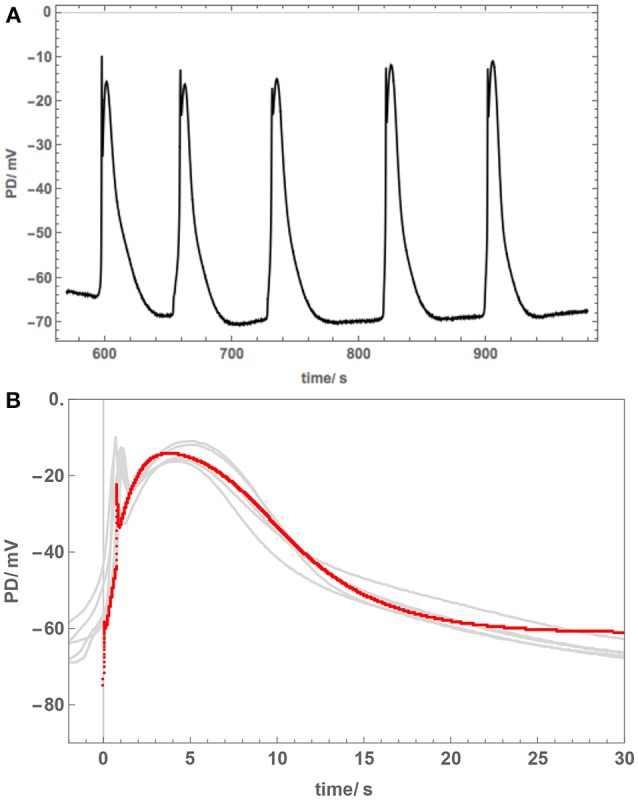
Cell 3: The overnight exposure to 50 mM NaCl can have varying effects. This cell became depolarized and cascade of spontaneous APs was recorded **(A)**. **(B)** The APs are similar to each other, but very different to those in Cells 1 and 2 (Figure 5). The model fit is shown as a red line. For parameters, see Table 2. Note prompt spikes at the start of each AP.

However, Cell 3, which was also treated overnight in 50 mM NaCl APW, exhibited different AP shape (Figure 6). This cell was also strongly depolarized and produced a cascade of spontaneous APs (Figure 6A). The shapes of these APs were similar to each other and prolonged compared to those in APW (Figure 6B), but quite different in shape to APs in Figure 5 and to saline APs in *Chara*. The spontaneous generation of the APs revealed sharp prompt spike at the beginning of each AP (this feature is difficult to resolve from the imposed stimulating depolarization). The spike is quite prominent in the more detailed plot of Figure 6B.

The initial modeling revealed that Resting PD before an AP is often more hyperpolarized than after excitation (see the shading in Figure 5A and Table 2B). This effect was previously masked by the averaging of the APs (Figure 2B). The change of the membrane PD was modeled by a transient inhibition of the proton pump due to the increase of the Ca^2+^ concentration in the cytoplasm (Methods and Discussion). The best agreement with the data was obtained by linearly decreasing (and sometimes subsequently increasing) pump parameter κ_oi_. The decrease was started between 0.5 and 5 s of the AP and stopped and sometimes reversed between 9 and 12 s of the data (Figures 3B, 5). The simple linear changes in the parameter show small discontinuities in some fitted profiles (Green at 10 s, Magenta at 8 s and Red at 5 s, Figure 5A). The difference of the pre- and post- membrane PD became greater with exposure to saline (compare Magenta, Red, and Orange AP data in Figure 5) until the pump was inhibited and the membrane PD was the same before and after excitation (yellow AP profile, Figure 5). The changes in the pump contribution to membrane PD at the time of excitation in the AP sequence in Figure 5 are shown in Figure 3B.

The model, as described in the Methods, was able to simulate most of the main features of the data in APW, Sorbitol and 50 mM NaCl APW (for parameters see Table 2). The fitted APs are shown as black lines in Figure 5A. The AP widened slightly upon exposure to 90 mM Sorbitol APW, but the parameters of the model are very similar to those in APW. The immediate exposure to 50 mM NaCl moved the AP peak to more negative level (Figure 5B) and produced slight widening of the early repolarizing phase. The model could only approximate the sharp return to steady PD and this effect was more noticeable as the cell was exposed to saline medium (Magenta AP profile, Figure 5A). In APs in APW, Sorbitol, and early NaCl exposure the membrane PD appears to overshoot the longer term post AP resting level (Figure 5B). This effect may be due to the partial inhibition of the proton pump by the high Ca ^2+^ in the cytoplasm (Figure 3B). A more detailed measurements will be done in future of the post-AP membrane PD and conductance for more accurate modeling. The Parameters p_1_′ and k_−3_ decreased with exposure to saline. This AP widening became more marked at 30 min, and at 60 min, the AP shape developed a long plateau. This transition in AP shape was very difficult to accommodate by the model and was not observed in *Chara*, where the AP form progressed directly to the flat plateau stage. A fit was achieved by decreasing the G_Cl, max_ parameter exponentially for the first 5 s of the excitation (Table 2). Similar low G_Clmax_ was observed after the overnight exposure of Cell 2 (Table 2).

The different response of the AP to overnight saline exposure in Cell 3 was reflected in higher parameter values of p_1_′ and unusually large p_2_′ (Table 2). Cell 3 illustrates a different saline AP shape to that of *Chara* that might be part of salt tolerance strategy.

### The Effect of 100 mM NaCl on AP Components Across Plasma and Tonoplast Membranes

**Figure 7:**
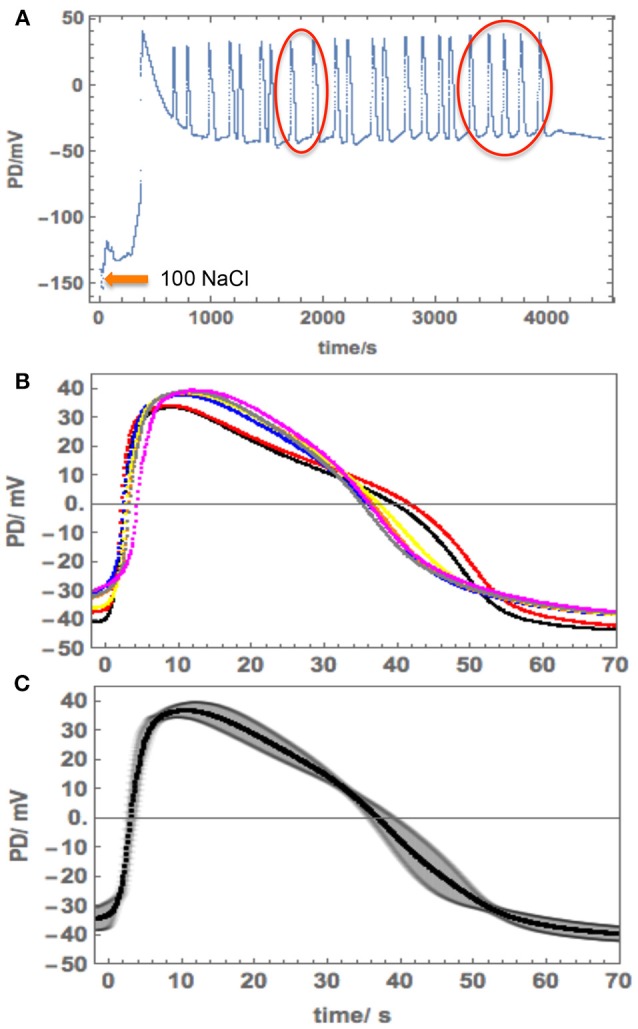
Repetitive APs in 100 mM NaCl APW in Cell 4. **(A)** The cell was exposed to 100 mM NaCl APW after pre-conditioning in 180 mM Sorbitol APW. Note the long timescale. **(B)** More detail of the selected APs (red circles in A). Note the AP timescale and amplitude. **(C)** The average AP with the standard deviation shown as the shaded area.

The salinity of 100 mM NaCl is limiting to *Nitellopsis* survival (Winter et al., 1999). A range of responses was observed upon cell exposure. Cell 4 was exposed to 100 mM NaCl after preconditioning in 180 mM Sorbitol APW. The membrane PD depolarized rapidly (Figure 7A), triggering 22 spontaneous APs. As the PD measuring electrode was in the vacuole, we expected to see excitation across both membranes. However, the detailed shape of the repetitive APs corresponded to the tonoplast AP. The shapes of all the APs were similar and we chose 7 APs (red circles in Figure 7A) to compute an average (Figures 7B,C).

The tonoplast AP shape in 100 mM NaCl APW was compared to the APW tonoplast AP obtained by subtraction of the plasma membrane AP from the AP across both membranes (Figure 4, orange line with orange shading depicting SD). To obtain the saline tonoplast PD alone, the PD across the plasma membrane was approximated as −55 mV, which gave similar tonoplast Resting PD to that in APW. Cell 4 provides a rare example of spontaneous tonoplast only APs.

**Figure 8:**
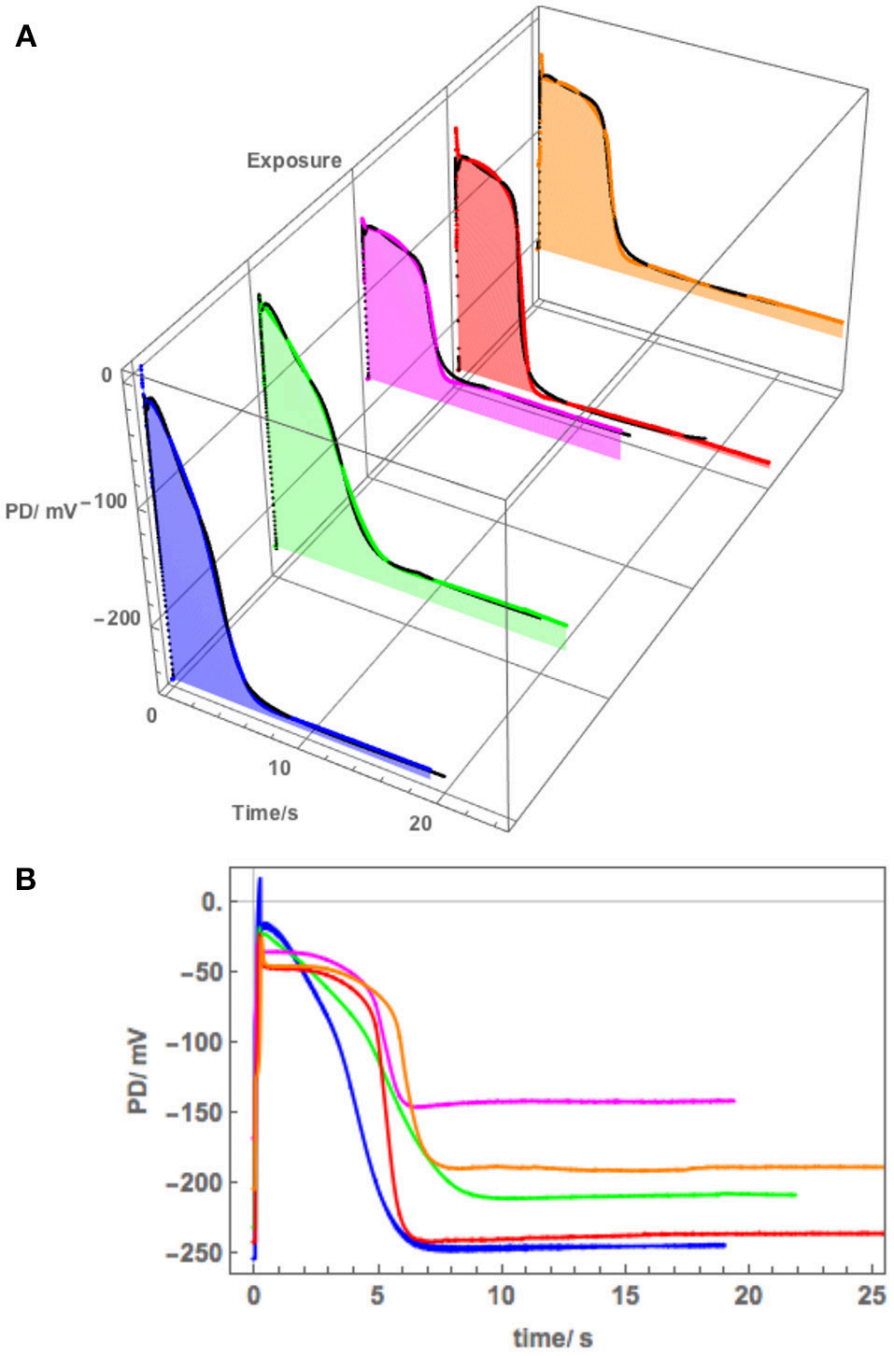
**(A)** The effect of 180 mM Sorbitol and 100 mM NaCl on the AP across the plasma membrane (Cell 5): blue–APW, green–Sorbitol, magenta−100 mM NaCl for 15 min, red−100 mM NaCl for 30 min, orange−100 mM NaCl for 60 min. The shading of each profile starts from the resting PD before excitation. The black lines show the model fit. The parameters can be found in Table 3. **(B)** Same AP sequence superimposed.

The membrane PD for Cell 5 was measured with the electrode in the cytoplasm. This cell exhibited very negative Resting PD in both APW and 180 mM Sorbitol APW (Table 3B). The exposure to 180 mM Sorbitol APW caused a greater widening of the AP than 90 mM solution with increases in p_1_′ and p_2_′ parameters (Table 3A and Figure 8). The vigorous pump action seemed to have protected the cell from strong effects of salinity on the AP form, which did not alter as much as in Cells 1 to 3 in 50 mM NaCl APW (compare Figures 5, 6, 8). The pump inhibition at the time of the AP was also less marked (see black fitted lines in Figure 8A). Cell 5 provides a good example of the saline AP response in hyperpolarized cell with a strong proton pump action.

**Figure 9:**
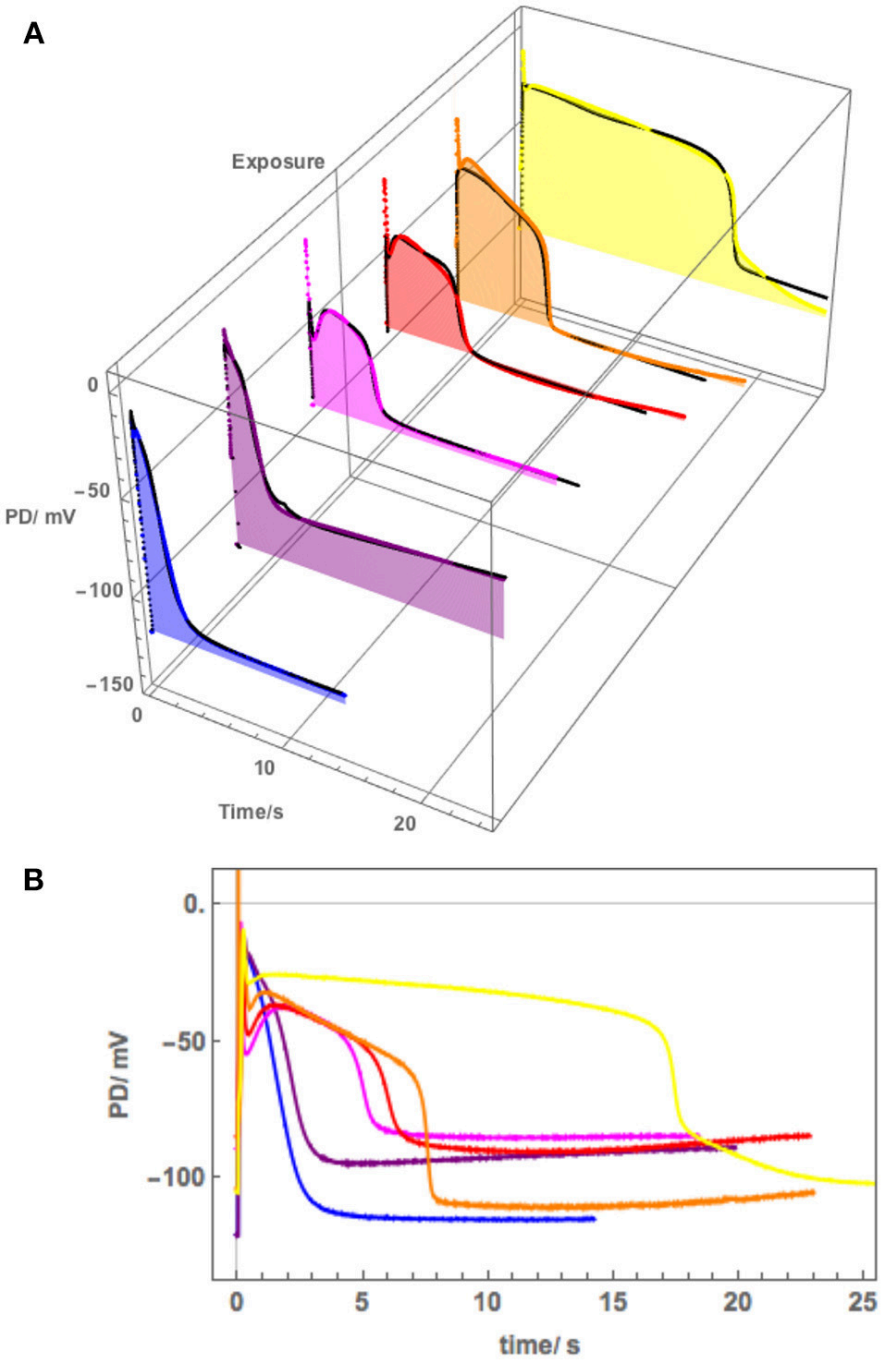
**(A)** The effect of 100 mM NaCl on the AP across the plasma membrane (Cell 6): blue–APW, purple−100 mM NaCl just on, magenta−100 mM NaCl for 15 min, red−100 mM NaCl for 30 min, orange−100 mM NaCl for 60 min, yellow−100 mM NaCl for 90 min. The shading of each profile starts from the resting PD before excitation. The black lines show the model fit. The parameters can be found in Table 4. **(B)** Same AP sequence superimposed.

Cell 6 exhibited depolarized Resting PD in APW and the AP shape responded much more strongly to exposure to 100 mM NaCl APW (Figure 9). The cell was preconditioned in 180 mM Sorbitol APW, but APs were not measured. The pump inhibition occurred at the time of the AP, when the cell was just exposed to saline APW (Purple AP profile, Figure 9A). In subsequent APs the membrane was too depolarized and the pump was not included in the modeling. There was only a small difference in the pre- and post- excitation membrane PD (Table 4B). Similarly to the effects of 50 mM NaCl APW, the AP peak became more negative and duration increased (Figure 9). The main parameters affected were p_1_′ (strong decrease), p_2_′ (decrease), and k_−3_ (decrease) (Table 4). Cell 6 supports the modeling strategy of partial proton pump inhibition by the AP, as APs with depolarized pre-excitation PD did not show decrease in post excitation PD level (Magenta, Red, Orange, and Yellow profiles in Figure 9B). Interestingly, very strong pump action (Cell 5) or no pump action (Cell 6) can have similar effect on the difference in pre and post excitation resting PD (compare Figures 8, 9). However, most hyperpolarized cells did show a small drop in the post-excitation resting PD.

## Discussion

*Nitellopsis obtusa* is native to deep lakes of Europe and Asia, but recently has been colonizing more polluted and shallow water habitats and became invasive in the Great Lakes of North America (Beilby and Casanova, 2014; Boissezon et al., 2018). It can survive in slightly brackish conditions (50–100 mM NaCl, Winter et al., 1999). It is not clear, whether this tolerance of brackish media contributes to *Nitellopsis* success in being American invader, as the Great Lakes are fed by freshwater rivers and have no direct contact with the ocean. However, salting of the roads in the winter and disturbance of the riverbeds might produce temporary saline areas in the lakes.

The initial experiments found that the *Nitellopsis* plasma membrane AP in the APW is ~2x the duration of the *Chara* plasma membrane AP under the same conditions, and the AP form is quite variable (Figure 2B). To fit the Thiel-Beilby model (Beilby and Al Khazaaly, 2016, 2017) the parameters involving both the Ca^2+^ release from, and the re-sequestration into, internal stores were adjusted. While the differences in most parameter values were small, k_−3_ parameter was consistently an order of magnitude larger (compare the *Chara* and *Nitellopsis* columns in Table 2A). The Cl^−^ channel activation rate constant, k_a_, was also increased (Table 2A). However, good fit was only obtained by changing the Hill coefficient n (Figure 3A). In the model (see section Materials and Methods), the Hill equation is used to quantify the action of the Ca^2+^ pumps re-sequestering Ca^2+^ into internal stores: $\frac{p1^{{}^{\prime }}C^{n}}{{p2^{\prime }}^{n}+C^{n}}$ (where C is the Ca^2+^ concentration in the cytoplasm). The Hill coefficient n provides a measure of substrate co-operativity in binding to the pump protein. For *n* > 1, there might be more than one substrate binding sites on the protein (two or more binding sites in a protein monomer or assembly of multiple subunits with a single substrate binding site**)** and binding of one substrate facilitates the binding of another substrate to the protein. For *n* = 1 the Hill equation becomes the Michaelis-Menten equation with no cooperativity. *Chara* AP was modeled using Hill equation with *n* = 2, resulting in a steep repolarization (see *Chara* AP in Figure 2B). The *Nitellopsis* AP required *n* = 1, with much more gradual repolarization (average *Nitellopsis* AP in Figure 2B), suggesting differences in the Ca^2+^ pumps on the internal stores in the two Characeae.

The vacuolar *Nitellopsis* AP exhibits quite large tonoplast component, compared to *Chara australis* (see Figures 1, 4 of Findlay and Hope, 1964). As the AP forms are quite variable, the resulting average tonoplast AP has large error bars associated with it. Findlay (1970) measured the tonoplast AP directly in *Nitellopsis obtusa* with range of results with the Resting PD between +10 to + 25 mV and variation in shape, with peak PD of up to + 70 mV (see Figure 2 of his paper). In our hands the average tonoplast Resting PD was +14 mV and the peak of the tonoplast AP was +133 mV. Even with the large SD of ± 30 mV, the amplitude of the tonoplast AP in our cells was somewhat larger than that observed by Findlay (1970) with a longer “tail.”

Because of this large tonoplast AP component, it was necessary to investigate the effect of salinity on the AP form across the plasma membrane alone. A sequence of APs (Figure 5) shows that both 90 mM Sorbitol APW and 50 mM NaCl prolong the AP shape in a similar manner to salt sensitive *Chara* (Beilby and Al Khazaaly, 2016, 2017). As *Nitellopsis* survives at this salinity, this result is surprising. The change to prolonged “rectangular” AP is more gradual in *Nitellopsis*. This is beautifully illustrated by the Orange AP profile in Figure 5, where a transition stage to the long plateau was captured and was modeled by a decrease in G_Cl, max_ (Table 2B). In terms of the model, same parameters are affected by salinity as in *Chara* to produce the “rectangular” AP: p_1_′ and p_2_′ decrease, while k_−3_ increases (Beilby and Al Khazaaly, 2017). However, in *Chara* the changes in the AP shape (and hence model parameter values) appear more at random, sometime exhibiting wide AP upon saline exposure and narrow APs after many minutes in saline (see, for instance, Figure 5 of Beilby and Al Khazaaly, 2017).

The AP shapes of the spontaneous AP cascade in Cell 3 were surprising, as they were quite different to rectangular APs, despite the cell overnight exposure to 50 mM NaCl APW. The gradual repolarization phase was reflected by increase in the p_2_′ parameter. Despite depolarized Resting PD, perhaps this AP shape represents a recovery, as *Nitellopsis* can grow in brackish conditions. Experiments following the cell exposures to longer periods of 50 mM NaCl APW will be necessary. The prompt initial spike observed in the APs (Figure 6B) is similar to that in *Chara* (Beilby and Al Khazaaly, 2016, 2017). Berestovsky and Kataev (2005) recorded Ca^2+^ transient currents in the *Chara* excitation. Thus, *Nitellopsis* AP confirms the existence of an early fast “priming” inflow of Ca^2+^ across plasma membrane, which mediates the steep depolarization phase of the characean AP.

As 100 mM NaCl is a long term limiting salinity level for *Nitellopsis* survival, especially if the Ca^2+^ concentration in the medium is low (Katsuhara and Tazawa, 1988; Winter et al., 1999), we expected to see a stronger effect on the AP. Once again, *Nitellopsis* had some surprises in store. Cell 4 provided an unexpected result: spontaneous repetitive APs due to sudden resting PD decline upon exposure of the cell to 100 mM NaCl APW (Figure 7A). Initially, this behavior seemed similar to that of *Chara australis* in 100 mM NaCl APW (Figure 2f of Shepherd et al., 2008). However, the detailed shape, duration and positive peak PD of the APs suggested that we are seeing repetitive tonoplast APs (Figures 7B,C). The observations of the tonoplast AP without accompanying plasma membrane AP are quite rare, but Findlay (1962) measured repetitive tonoplast only APs in *Nitella* sp., which also has a large tonoplast AP component. In Figure 4 we compare the average saline tonoplast AP (after adding 55 mV, as the estimated PD across the plasma membrane, orange line), the APW subtracted average tonoplast AP (green points) and the APW AP across plasma membrane (blue points) and both membranes (red points). The data are not sufficient to determine if the tonoplast AP is affected by salinity, but provides a starting point for future experiments.

Cell 5 exhibited a very negative Resting PD, due to strong action of the proton pumps. The exposure to 180 mM Sorbitol APW did widen the AP duration and subsequent 100 mM NaCl APW did affect the AP shape (Figure 8), but not to the same extent as the half saline concentration of 50 mM NaCl APW (compare to Figure 5). This response was surprising, but underlined the importance of the role of the proton pump in salt tolerance. To confirm the hypothesis, Cell 6 was depolarized in APW (suggesting weak proton pump action) and the 100 mM NaCl APW had strong effect on the AP, extending the duration nearly to 20 s in 90 min of saline exposure (Figure 9).

Smith and Beilby (1983) measured transient membrane conductance decrease following the AP in *Chara* and suggested that the proton pump was fully or partially inhibited by the rise in cytoplasmic Ca^2+^ concentration. In *Chara* the inhibition reached a maximum many s after the AP conclusion. *Nitellopsis* exhibits similar effect, and the long-term post AP conductance changes will be investigated in future experiments. As the *Nitellopsis* AP is longer than that of *Chara* in APW, it was instructive to include the pump inhibition in the model, where it improved the fit at the AP conclusion (see, for instance, the Green, Magenta, and Red profiles in Figure 5A) The proton pump inactivation has been observed and modeled in higher plants (Sukhov and Vodeneev, 2009).

Similarly to *Chara*, the *Nitellopsis* AP peak potential remained relatively constant, despite the increase of Cl^−^ concentration in the medium from 1.3 mM in APW to 50 and 100 mM in the saline media. *Chara australis* has a low concentration of cytoplasmic Cl^−^ (Coster, 1966). In the model the cytoplasmic Cl^−^ was equilibrated with the medium (Teakle and Tyerman, 2010; Beilby and Al Khazaaly, 2016). The cytoplasmic Cl^−^ concentration is higher in *Nitellopsis* and does not change during short exposures to 100 mM NaCl (Katsuhara and Tazawa, 1986 and Tables 2–4). As in the *Chara* AP model, the maximum Cl^−^ conductance, G_Cl, max_, increased upon saline exposure (Tables 2–4). However, the *Nitellopsis* AP peak became gradually more negative with saline exposure and G_Cl, max_ decreased. This effect was particularly marked in the 60 min saline exposure of Cell 1 (Figure 5 and Table 2). We speculate that the conductance of the Cl^−^ channels is enhanced by the pre-excitation hyperpolarized membrane PD and by greater Cl^−^ concentrations in the medium (Beilby and Al Khazaaly, 2016). Katsuhara and Tazawa (1986) also measured cytoplasmic K^+^ concentration drop upon exposure to 100 mM NaCl and their results were utilized in the model (Tables 2–4).

## Conclusions and Future Perspectives

The AP form in *Nitellopsis* is longer in freshwater conditions, compared to that of *Chara*, with more gradual repolarization. The longer re-sequestration of Ca^2+^ could be due to an older, possibly more primitive, Ca^2+^ pumps. It will be interesting to compare the genetic fingerprints of the pump/s with those of other Characeae, higher plants and the chlorophyte algae, living in seawater. The form of the plasma membrane AP is prolonged by the exposure to saline, but the changes are more gradual than in *Chara*, allowing more insight into the mechanism (for instance the change in G_Cl, max_ parameter after exposure to 50 mM NaCl in Cell 1, or the changes of the proton pump rate at the time of excitation). It is possible that the cells recover the original AP form after prolonged exposure to brackish conditions. Some cells experience tonoplast APs only, where the Cl^−^ might be easier to recover by the vacuole, thus providing adaptation to brackish media. As in *Chara*, the proton pump is transiently inhibited by the high cytoplasmic Ca^2+^ and gradually declines in saline media. However, if the cells are very hyperpolarized at the start of the experiment, the pump inhibition both by the AP and by the saline medium is mitigated. Future experiments will investigate electrophysiological characteristics of long-term survival and growth of *Nitellopsis* in brackish media with 20–50 mM NaCl to resolve in detail this partial step toward salt tolerance.

Is our research relevant to land plants? In recent years APs became important as multifunctional signals in excitable tissues of higher plants (Sukhov and Vodeneev, 2009). Initially APs were thought to only occur in sensitive plants, such as *Mimosa* or *Dionaea*. However, APs are now detected in many land plants, including food crops (barley, rye, maize, cucumber, tomato, peas, and beans), as well as in the model plant *Arabidopsis*. AP propagation affects vital plant functions, such as photosynthesis, gene expression, production of phytohormones, or stomatal movements. APs signal many types of biotic and abiotic stress (Yan et al., 2009; for review see Sukhov and Vodeneev, 2009; Sukhov et al., 2011). The detailed knowledge of salinity effect on the AP mechanism is likely to be applicable to cells in plant roots, which come into contact with changing levels of salt (see Canales et al., 2018 for root to shoot electrical signaling).

## Data Availability

The trends of parameters p_1_′, p_2_′ and k_−3_ for the Cells 1 - 6 with different treatments have been graphed on MJB website http://newt.phys.unsw.edu.au/~mjb/APproj.html

## Author Contributions

VK, IL, and VP performed the experiments, while MB analyzed the data and fitted the Thiel-Beilby model. All authors listed contributed to writing the paper and approved it for publication.

### Conflict of Interest Statement

The authors declare that the research was conducted in the absence of any commercial or financial relationships that could be construed as a potential conflict of interest.
